# Global Trends in the Incidence of Anxiety Disorders From 1990 to 2019: Joinpoint and Age-Period-Cohort Analysis Study

**DOI:** 10.2196/49609

**Published:** 2024-01-29

**Authors:** Huiru Cao, Yang Wu, Hui Yin, Yanqi Sun, Hui Yuan, Mengjun Tao

**Affiliations:** 1 Department of Gastroenterology The First Affiliated Hospital of Wannan Medical College Wuhu China; 2 Department of Health Management Center The First Affiliated Hospital of Wannan Medical College Wuhu China; 3 Department of Hospital Infection The First Affiliated Hospital of Wannan Medical College Wuhu China; 4 Department of Prevention and Health Care People's Hospital of Rizhao Rizhao China; 5 School of Public Health Wannan Medical College Wuhu China

**Keywords:** age-period-cohort analysis, anxiety disorders, incidence, disability-adjusted life-years, DALYs, joinpoint regression model, prediction

## Abstract

**Background:**

Anxiety disorders (ADs) are the most common mental illness with high prevalence, chronicity, and comorbidity. Despite rapid economic and cultural development, the global incidence of ADs continues to increase, with predominance in male individuals.

**Objective:**

To address the above issues, we analyzed the dynamic trends of the global incidence and disease burden of ADs from 1990 to 2019 and their different effects on age, period, and birth cohort and predicted the future trend of AD incidence.

**Methods:**

The data were obtained from the Global Burden of Disease study in 2019. A joinpoint regression model was used to calculate the annual percent change in AD incidence, and age-period-cohort analysis was used to estimate the independent effects of age, period, and cohort. Nordpred age-period-cohort analysis was used to predict the incidence of ADs from 2020 to 2044.

**Results:**

The age-standardized incidence rate of ADs increased by 1.06% for both sexes, and the age-standardized disability-adjusted life-year (DALY) rate (ASDR) decreased by 0.12%. Joinpoint regression indicated that increments in average annual percent changes in the age-standardized incidence rate (0.068 vs 0.012) and ASDR (0.035 vs –0.015) for ADs globally were higher among male individuals than female individuals. The age-period-cohort analyses revealed that the relative risk (RR) of the incidence and DALYs of ADs among people of different sexes increased with age in adolescence and middle age and then decreased. For the period effect, the RR of incidence decreased, whereas the RR of DALYs increased in both sexes. Moreover, the RR of the incidence gradually increased and DALYs slowly decreased with birth year for both male and female individuals. New cases of ADs in male individuals are predicted to increase in the coming 25 years.

**Conclusions:**

This study provided the changing trend of the global incidence and disease burden of ADs in the past 3 decades, indicating that early prevention and effective control cannot be ignored. We analyzed the age-period-cohort effect of potential trends in ADs and predicted future incidence trends. The results suggest that we should take active intervention measures, focusing on high-risk groups and developing effective management and control policies to reduce the global burden of disease.

## Introduction

Anxiety disorders (ADs) form the most common group of mental disorders, which are characterized by excessive and enduring fear, anxiety, or avoidance of perceived threats and can also include panic attacks [1], with generalized ADs being the most common AD seen in primary care [2]. This is based on the *Diagnostic and Statistical Manual of Mental Disorders, Fifth Edition* (DSM-5) and the *International Classification of Diseases, 11th Edition* (ICD-11) diagnostic systems, which categorize ADs based on key symptoms at 3 levels such as cognitive, emotional, and somatic, including separation anxiety, selective mutism, specific phobias, social AD, panic disorder, agoraphobia, and generalized AD [1,3]. The lifetime prevalence rates of AD have reached 34% [4], and ADs generally start in childhood or adolescence [5,6]. Most patients with ADs often have comorbid ADs and other mental disorders, especially depression [7] and somatic disorders such as heart disease, hyperthyroidism, asthma, and epilepsy [8-10]. Importantly, ADs are 2 times more prevalent among female individuals than male individuals [11]. The high prevalence, chronicity, and comorbidity of ADs led the World Health Organization to rank ADs as the ninth leading health-related cause of disability [12]. ADs not only seriously affect patients’ daily functioning and quality of life but also impose a heavy burden on society and account for 3.3% of the global burden of disease (GBD) [13,14].

According to the large-scale World Mental Health Surveys, the prevalence of ADs was highest in high-income countries, especially in Australia, the United States, and European countries [15,16]. Estimated from the 2010 cost model, over €74 billion (US $81.64 billion) was largely due to indirect costs such as disability for 30 European countries [13]. Even if there is insufficient evidence to show that the prevalence of ADs is increasing, the burden of disease cannot be ignored. ADs are still an important global public health problem.

At present, there are relatively few studies on the global trend in the prevalence of ADs, and they use only traditional descriptive analysis of age-specific incidence or mortality data at different times, which cannot eliminate or control for the interaction among age, period, and cohort factors. The joinpoint regression (JPR) model is mainly used to analyze the time trend of incidence and mortality and to analyze the burden of disease, which can better reflect the change in the epidemic trend and its impact [17,18]. The age-period-cohort model improved the traditional descriptive analysis method to estimate the risk of disease incidence or mortality and its trend while adjusting for age, period, and cohort [18,19].

Therefore, this study analyzed data from the GBD 2019 study. The JPR model was used to explore the temporal trends in the incidence and disability-adjusted life-year (DALY) rates of ADs globally and to explore the net age, period, and cohort effects with the age-period-cohort model. Meanwhile, the prediction of the incidence of ADs in the next 25 years based on these data can provide a scientific basis for the evaluation of AD prevention and treatment to reduce the disease burden caused by ADs.

## Methods

### Data Sources

Data on AD trends during 1990-2019 were retrieved from the World Health Organization GBD estimates. GBD data are derived from private and public organizations worldwide that collect data through surveys, reports, scientific literature, censuses, and other methods. The study has provided improved standardized methods and a comprehensive assessment of incidence and DALYs for 369 diseases and injuries and 87 risk factors in 204 countries and territories. Further details of the general methodologies for the GBD have been described elsewhere [20-22], and data and the protocol for the 2019 GBD can be accessed through the Global Health Data Exchange GBD Results Tool. All GBD 2019 analyses adhered to the Guidelines for Accurate and Transparent Health Estimates Reporting [23].

### Ethical Considerations

This study used data from the GBD Study 2019, which was approved by the institutional review board of the University of Washington. Original data were collected with informed consent from study participants or with a waiver from the institutional review board. As this was a secondary analysis of publicly available data, no further review by an institutional review board was required following the data use agreement of The Institute for Health Metrics and Evaluation.

### Statistical Analysis

#### Overall Temporal Trends Analysis of Global

Data on the global and regional incidence of ADs from 1990 to 2019 were extracted from the GHDx database, including crude incidence (CIR), DALYs, age-standardized incidence, and age-standardized DALYs of ADs in the all-age group. Data are reported as estimates with 95% uncertainty intervals (UIs). Age-standardized incidence rates (ASIRs) and age-standardized DALY rates (ASDRs) were used to assess the temporal trend of the global incidence of ADs from 1900 to 2019. The ASIR and ASDR in the GBD database were standardized based on 2019 global population data [20].

#### JPR Analysis

JPR model analysis was applied to estimate the trends in AD prevalence from 1990 to 2019. As proposed by Kim et al [24], joinpoint analysis can automatically divide longitudinal variations into different segments by segmented regression and identify the segment trends with statistical significance. Regression fitting was performed on the natural logarithm of the prevalence and mortality rate in different segments, and then the annual percent changes (APCs) and their 95% CIs were calculated for each period. The global trend was described by the average annual percent changes (AAPCs). The APCs and AAPCs were considered statistically significant by nonoverlapping 95% CIs and *P*<.05 compared to the null hypothesis of no variation. We applied the JPR program (version 4.8.0.1) from the Statistical Research and Applications Branch of the Surveillance Research Program of the US National Cancer Institute. 

#### Age-Period-Cohort Analysis

The age-period-cohort model was used to estimate the relative risk (RR) of the population in a given year and the cumulative health risk since birth. The model allowed analysis of the independent effects of age, period, and cohort on temporal trends in incidence and DALY rate due to ADs and other forms of ADs. The age-period-cohort model provides a useful parametric framework that can complement standard nonparametric descriptive methods. This analysis model has been used and recognized in many previous studies [18,19]. In this model, the data collected were stratified into age groups of 5 consecutive years and periods of 5 consecutive years. The incidence rate and DALY rate of AD in 5 consecutive age groups (0-5 years), 5 consecutive periods (1990-1994 to 2015-2019), and 5 consecutive birth cohorts (1990-2015) were recorded. Age-period-cohort analysis using the intrinsic estimation method provided coefficient estimates for age, period, and cohort effects. These coefficients are converted to exponential values [exp (coefficient)=ecoefficient], representing the RR of incident AD and AD-related DALY rates for a specific age, period, or birth cohort relative to the mean for all ages, periods, or birth cohorts [25]. Age-period-cohort analysis was performed using STATA software (version 15.0; StataCorp).

#### Incidence Rate Predicted

This study predicted the incidence rate of ADs from 2020 to 2044 by running a Nordpred age-period-cohort analysis by sex using the *Nordpred* package in R (R Foundation for Statistical Computing), accounting for changing rates and changing population structures. This method has been fully demonstrated and recognized in previous studies [26].

## Results

### Global Trends in the ASIRs and ASDRs of ADs From 1990 to 2019

**Figure 1:**
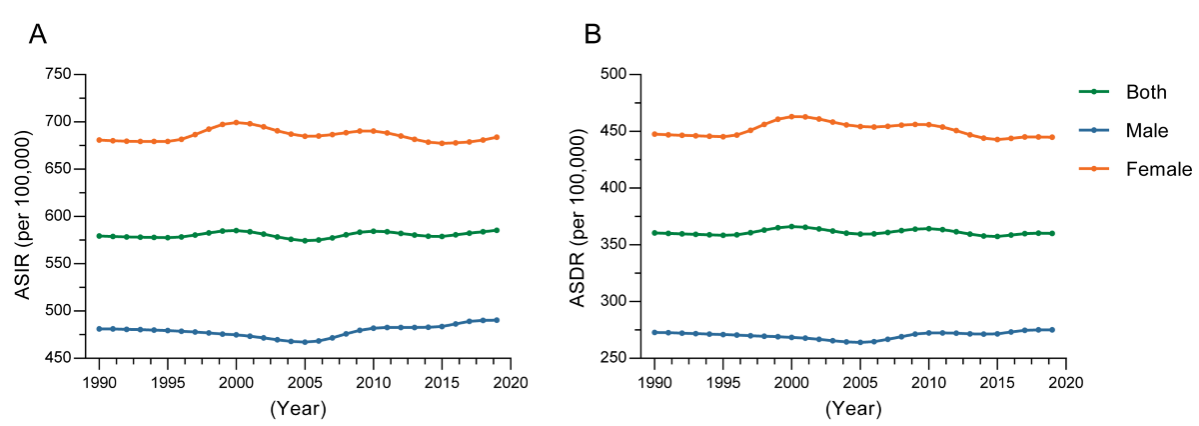
Trends in the sex-specific incidence and DALY rate of ADs globally during 1990-2019. (A) ASIR of ADs and (B) ASDR of ADs. AD: anxiety disorder; ASDR: age-standardized DALY rate; ASIR: age-standardized incidence rate; DALY: disability-adjusted life-year.

The ASIR of ADs showed a slightly fluctuating upward trend, increasing from 579.30 (95% UI 467.81-702.90) per 100,000 in 1990 to 585.45 (95% UI 474.21-709.53) per 100,000 in 2019, with an increase of 1.06%. The growth rate of the ASIR was higher in male individuals than female individuals, although the incidence rate for female individuals is about 1.4 times higher than that of male individuals. Among them, the ASIR increased by 1.92% in male individuals and 0.43% in female individuals (Figure 1A).

The trend of ASDR for ADs was relatively stable from 360.55 (95% UI 250.82-492.46) per 100,000 in 1990 to 360.12 (95% UI 248.60-494.44) per 100,000 in 2019, decreased by 0.12%. The ASDR increased by 0.90% in male individuals and decreased by 0.6% in female individuals (Figure 1B).

### JPR Analysis

**Figure 2:**
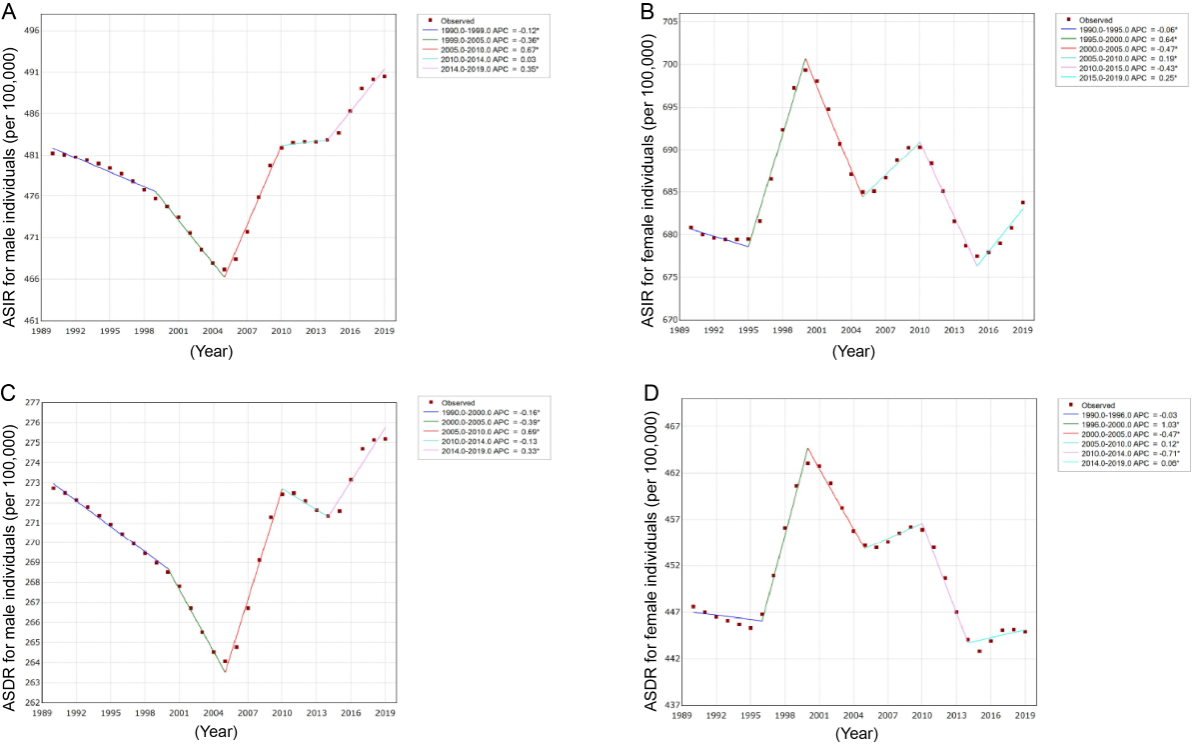
Joinpoint regression analysis of the sex-specific age-standardized incidence and disability-adjusted life years rate for ADs in global from 1990 to 2019. (A) Age-standardized incidence rate (ASIR) for males; (B) Age-standardized incidence rate (ASIR) for females; (C) Age-standardized DALYs rate (ASDR) for males; (D) Age-standardized DALYs rate (ASDR) for females. For a higher-resolution version of this figure, see Multimedia Appendix 1.

The joinpoint model was applied to divide the temporal trends in AD incidence into several segments and estimate the APCs by sex. As displayed in Table 1 and Figure 2A, the ASIR of ADs among male individuals declined first (APCs_1990-1999_=−0.12%), significantly increased (APCs_2005-2010_=0.67%), and then increased thereafter (APCs_2010-2014_=0.03% and APCs_2014-2019_=0.35%). In contrast, the ASIR underwent 3 increases (APCs_1995-2000_ =0.64%, APCs_2005-2010_=0.19%, and APCs_2015-2019_=0.25%) and 3 declines (APCs_1990-1995_=–0.06%, APCs_2000-2005_=–0.47%, and APCs_2010-2015_=–0.43%) among female individuals, as shown in Figure 2B. Moreover, the trend in the ASDR was consistent with the change in the ASIR (Table 1; Figure 2C and D).

Over the entire study period, the ASIRs of ADs were 0.068% (95% CI 0.35-1.0) among male individuals and 0.012% (95% CI 0.8-1.0) among female individuals (Table 1; Figure 2A and B).

**Table 1:** Joinpoint regression analysis of the sex-specific age-standardized incidence and DALY^a^ rate for anxiety disorders globally from 1990 to 2019.

Categories, sex, and period			APC^b^ (95% CI)
**Incidence**			
	**Male**		
		1990-1999	–0.123 (–0.153 to –0.094)^c^
		1999-2005	–0.363 (–0.435 to –0.292)^c^
		2005-2010	0.675 (0.572 to 0.777)^c^
		2010-2014	0.033 (–0.128 to –0.194)
		2014-2019	0.353 (0.280 to 0425)^c^
	**Female**		
		1990-1995	–0.060 (–0.095 to –0.025)^c^
		1995-2000	0.643 (0.593 to 0.693)^c^
		2000-2005	–0.469 (–0.518 to –0.419)^c^
		2005-2010	0.189 (0.139 to 0.238)^c^
		2010-2015	–0.475 (–0.376 to –18.609)^c^
		2015-2019	0.246 (0.196 to 0.296)^c^
**AAPC^d^ of incidence**			
	Male		0.068 (0.035 to 0.100)^c^
	Female		0.012 (–0.006 to 0.030)
**DALY rates**			
	**Male**		
		1990-1999	–0.158 (–0.183 to –0.132)^c^
		1999-2005	–0.387 (–0.491 to –0.283)^c^
		2005-2010	0.688 (0.583, 0.792)^c^
		2010-2014	–0.128 (–0.292 to 0.037)
		2014-2019	0.326 (0.252 to 0.400)^c^
	**Female**		
		1990-1996	–0.035 (–0.073 to 0.003)
		1996-2000	1.027 (0.914 to 1.140)^c^
		2000-2005	–0.470 (–0.540 to –0.400)^c^
		2005-2010	0.121 (0.050 to 0.192)^c^
		2010-2014	–0.714(–0.825 to –0.603)^c^
		2014-2019	0.063 (0.013 to 113)^c^
**AAPC of DALY rates**			
	Male		0.035 (0.001 to 0.070)^c^
	Female		–0.015 (–0.042 to –0.013)

^a^DALY: disability-adjusted life-year.

^b^APC: annual percent change.

^c^Changes that are statistically significant.

^d^AAPC: average annual percent change.

### Age-Period-Cohort Analysis

#### Age Effect

**Figure 3:**
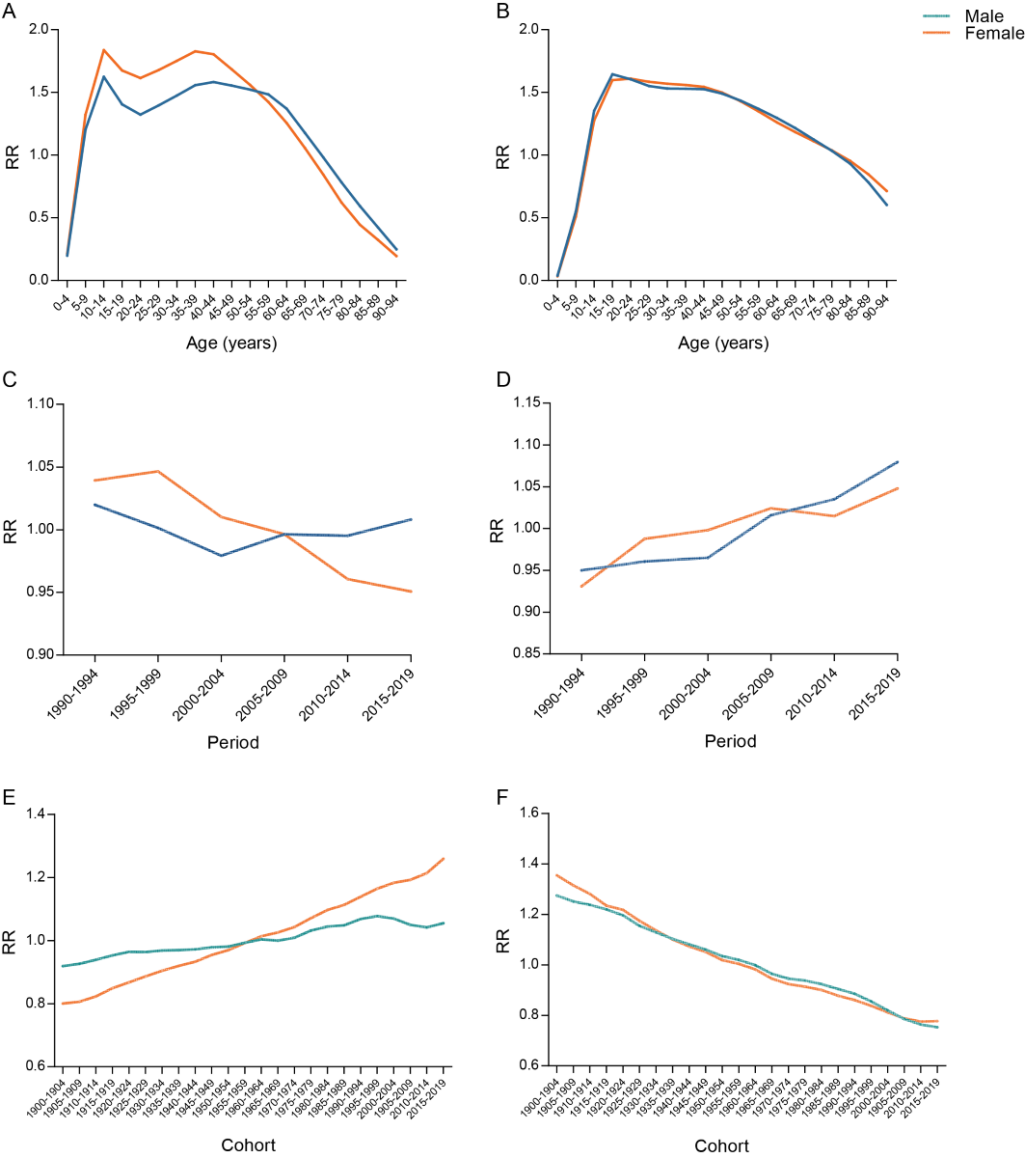
RRs of the incidence and disability-adjusted life-year (DALY) rate of anxiety disorders globally from 1990 to 2019 due to age, period, and cohort effects. (A) Age effects on incidence, (B) age effects on DALY rate, (C) period effects on incidence, (D) period effects on DALY rate, (E) cohort effects on incidence, and (F) cohort effects on DALY rate. RR: relative risk.

Table 2 and Figure 3A and B show the longitudinal age curve of the incidence and DALY rates of ADs. After adjusting for period effects, in the reference cohort, the incidence of ADs showed 2 upward and downward trends with age in the reference cohort, similar to an M-shaped curve. Among both male and female individuals, individuals aged 10-14 years had the highest incidence of ADs. The second peak occurred at ages 40-44 years for male individuals and at ages 35-39 years for female individuals. In addition, the DALY rate of ADs was highest at 15-19 years of age for male individuals and 20-24 years of age for female individuals, and then the DALY rate decreased with age.

**Table 2:** RRs^a^ of the incidence and disability-adjusted life-year (DALY) rates of anxiety disorders globally from 1990 to 2019 due to age, period, and cohort effects.

Factor		Incidence, RR (95% CI)		DALY rates, RR (95% CI)	
		Male	Female	Male	Female
**Age group (years)**					
	0-4	0.198 (0.180-0.218)^b^	0.214 (0.198-0.232)^b^	0.039 (0.029-0.052)^b^	0.035 (0.027-0.044)^b^
	5-9	1.204 (1.153-1.257)^b^	1.323 (1.274-1.374)^b^	0.550 (0.506-0.598)^b^	0.509 (0.476-0.544)^b^
	10-14	1.626 (1.565-1.688)^b^	1.838 (1.779-1.899)^b^	1.354 (1.273-1.439)^b^	1.276 (1.216-1.340)^b^
	15-19	1.405 (1.325-1.460)^b^	1.675 (1.622-1.730)^b^	1.645 (1.555-1.741)^b^	1.598 (1.528-1.671)^b^
	20-24	1.324 (1.273-1.376)^b^	1.615 (1.564-1.667)^b^	1.604 (1.518-1.695)^b^	1.610 (1.542-1.682)^b^
	25-29	1.397 (1.345-1.452)^b^	1.679 (1.627-1.732)^b^	1.552 (1.469-1.639)^b^	1.585 (1.519-1.654)^b^
	30-34	1.476 (1.421-1.533)^b^	1.752 (1.698-1.809)^b^	1.530 (1.450-1.615)^b^	1.569 (1.505-1.637)^b^
	35-39	1.558 (1.499-1.618)^b^	1.828 (1.770-1.888)^b^	1.529 (1.450-1.613)^b^	1.560 (1.497-1.626)^b^
	40-44	1.584 (1.523-1.646)^b^	1.805 (1.746-1.866)^b^	1.526 (1.447-1.609)^b^	1.544 (1.482-1.609)^b^
	45-49	1.554 (1.493-1.617)^b^	1.684 (1.626-1.745)^b^	1.492 (1.416-1.572)^b^	1.498 (1.439-1.560)^b^
	50-54	1.523 (1.462-1.585)^b^	1.559 (1.502-1.618)^b^	1.436 (1.364-1.512)^b^	1.431 (1.374-1.489)^b^
	55-59	1.484 (1.425-1.547)^b^	1.424 (1.369-1.480)^b^	1.368 (1.300-1.440)^b^	1.348 (1.296-1.403)^b^
	60-64	1.369 (1.313-1.428)^b^	1.256 (1.205-1.308)^b^	1.296 (1.232-1.363)^b^	1.261 (1.213-1.312)^b^
	65-69	1.177 (1.126-1.230)^b^	1.056 (1.010-1.103)^c^	1.215 (1.156-1.278)^b^	1.184 (1.139-1.231)^b^
	70-74	0.982 (0.937-1.029)	0.845 (0.806-0.886)^b^	1.125 (1.070-1.183)^b^	1.111 (1.069-1.154)^b^
	75-79	0.782 (0.744-0.823)^b^	0.662 (0.590-0.656)^b^	1.034 (0.983-1.089)	1.037 (0.998-1.079)
	80-84	0.595 (0.562-0.630)^b^	0.446 (0.420-0.474)^b^	0.931 (0.883-0.983)^d^	0.954 (0.917-0.994)^c^
	85-89	0.422 (0.395-0.451)^b^	0.323 (0.300-0.347)^b^	0.783 (0.739-0.830)^b^	0.846 (0.810-0.883)^b^
	90-94	0.250 (0.228-0.273)^b^	0.195 (0.177-0.215)^b^	0.602 (0.562-0.646)^b^	0.713 (0.679-0.749)^b^
**Period**					
	1994	1.020 (1.000-1.041)	1.039 (1.021-1.058)^b^	0.950 (0.926-0.975)^b^	0.931 (0.913-0.950)^b^
	1999	1.001 (0.981-1.022)	1.047 (1.028-1.065)^b^	0.961 (0.937-0.985)^d^	0.988 (0.969-1.007)
	2004	0.979 (0.960-0.999)^c^	1.010 (0.993-1.028)	0.965 (0.941-0.990)^d^	0.998 (0.979-1.017)
	2009	0.996 (0.977-1.017)	0.996 (0.979-1.014)	1.016 (0.991-1.041)	1.024 (1.005-1.044)^c^
	2014	0.995 (0.975-1.016)	0.961 (0.944-0.978)^b^	1.035 (1.009-1.061)^d^	1.015 (0.995-1.035)
	2019	1.008 (0.987-1.029)	0.951 (0.933-0.969)^b^	1.080 (1.053-1.107)^b^	1.048 (1.028-1.069)
**Cohort**					
	1900	0.919 (0.749-1.128)	0.800 (0.642-0.997)^c^	1.275 (1.095-1.485)^d^	1.355 (1.218-1.507)^b^
	1905	0.927 (0.816-1.052)	0.806 (0.704-0.924)^d^	1.251 (1.127-1.389)^b^	1.315 (1.220-1.417)^b^
	1910	0.939 (0.855-1.032)	0.823 (0.743-0.911)^b^	1.238 (1.138-1.347)^b^	1.218 (1.205-1.363)^b^
	1915	0.953 (0.883-1.028)	0.849 (0.783-0.920)^b^	1.220 (1.135-1.311)^b^	1.235 (1.170-1.303)^b^
	1920	0.964 (0.904-1.028)	0.867 (0.811-0.928)^b^	1.196 (1.121-1.277)^b^	1.218 (1.160-1.279)^b^
	1925	0.964 (0.910-1.020)	0.886 (0.836-0.940)^b^	1.155 (1.087-1.226)^b^	1.174 (1.122-1.229)^b^
	1930	0.968 (0.918-1.021)	0.904 (0.856-0.955)^b^	1.129 (1.063-1.198)^b^	1.136 (1.085-1.190)^b^
	1935	0.969 (0.921-1.020)	0.919 (0.873-0.968)^d^	1.103 (1.038-1.171)^d^	1.100 (1.050-1.153)^b^
	1940	0.972 (0.926-1.021)	0.933 (0.889-0.980)^d^	1.082 (1.018-1.149)^c^	1.073 (1.023-1.125)^d^
	1945	0.979 (0.933-1.026)	0.954 (0.911-0.999)^c^	1.060 (0.997-1.128)	1.051 (1.002-1.103)^c^
	1950	0.981 (0.936-1.027)	0.970 (0.929-1.013)	1.034 (0.972-1.101)	1.019 (0.970-1.070)
	1955	0.993 (0.949-1.039)	0.992 (0.952-1.034)	1.019 (0.957-1.086)	1.004 (0.956-1.055)
	1960	1.004 (0.961-1.049)	1.014 (0.975-1.054)	0.999 (0.937-1.064)	0.982 (0.934-1.032)
	1965	1.000 (0.958-1.044)	1.025 (0.988-1.064)	0.964 (0.905-1.028)	0.945 (0.899-0.994)^c^
	1970	1.009 (0.968-1.053)	1.043 (1.007-1.081)^c^	0.945 (0.887-1.008)	0.923 (0.878-0.971)^d^
	1975	1.032 (0.991-1.075)	1.071 (1.036-1.108)^b^	0.938 (0.880-1.000)^c^	0.914 (0.869-0.961)^b^
	1980	1.044 (1.004-1.086)^c^	1.097 (1.063-1.133)^b^	0.924 (0.886-0.985)^c^	0.900 (0.856-0.947)^b^
	1985	1.048 (1.009-1.089)^c^	1.113 (1.079-1.148)^b^	0.904 (0.846-0.966)^d^	0.878 (0.833-0.925) ^b^
	1990	1.068 (1.026-1.112)^d^	1.139 (1.102-1.177)^b^	0.885 (0.826-0.950)^d^	0.860 (0.814-0.909)^b^
	1995	1.078 (1.032-1.126)^d^	1.164 (1.123-1.207)^b^	0.855 (0.792-0.923)^b^	0.838 (0.788-0.890)^b^
	2000	1.070 (1.018-1.124)^d^	1.183 (1.135-1.232)^b^	0.819 (0.751-0.894)^b^	0.812 (0.758-0.870)^b^
	2005	1.050 (0.989-1.114)	1.193 (1.135-1.253)^b^	0.785 (0.704-0.876)^b^	0.788 (0.722-0.860)^b^
	2010	1.042 (0.957-1.133)	1.213 (1.131-1.302)^b^	0.764 (0.637-0.916)^d^	0.776 (0.670-0.898)^d^
	2015	1.055 (0.835-1.332)	1.259 (1.040-1.524)^c^	0.753 (0.355-1.597)	0.777 (0.423-1.427)
Deviance		0.073	0.067	0.042	0.062
AIC^e^		8.597	8.804	8.117	8.656
BIC^f^		–317.081	–317.523	–319.184	–317.839

^a^RR: relative risk.

^b^*P*<.001.

^c^*P*<.05.

^d^*P*<.01.

^e^AIC: Akaike information criterion.

^f^BIC: Bayesian information criterion.

#### Period Effect

Table 2 and Figure 3C and D show the estimated period effects by sex during the whole study period. Regarding the incidence, the period effects showed a downward trend from 1990-1994 to 2000-2004 for male individuals, with the period RR decreasing from 1.02 to 0.98 and then slightly increasing in 2015-2019. However, the period RR slightly increased from 1990-1994 to 1995-1999 for female individuals, and then it decreased. Regarding the DALY rate, the period RR showed similar increasing patterns for both sexes (increased by 13.63% for male individuals and 11.70% for female individuals).

#### Cohort Effect

The risk of ADs increased slowly with the year of birth for both sexes (Table 2; Figure 3E). Specifically, from the 1990-1994 to 2015-2019 birth cohorts, the RR of AD incidence substantially increased by 57.34% for female individuals. However, the cohort RR slightly increased by 17.32% from 1990-1994 to 1995-1999 for male individuals and then slightly fluctuated. Regarding the DALY rate, the period RR showed similar decreasing patterns for both sexes (decreased by 40.94% for male individuals and 42.64% for female individuals), as shown in Table 2 and Figure 3F.

#### Predicted Trends of ADs in 2020-2044

**Figure 4:**
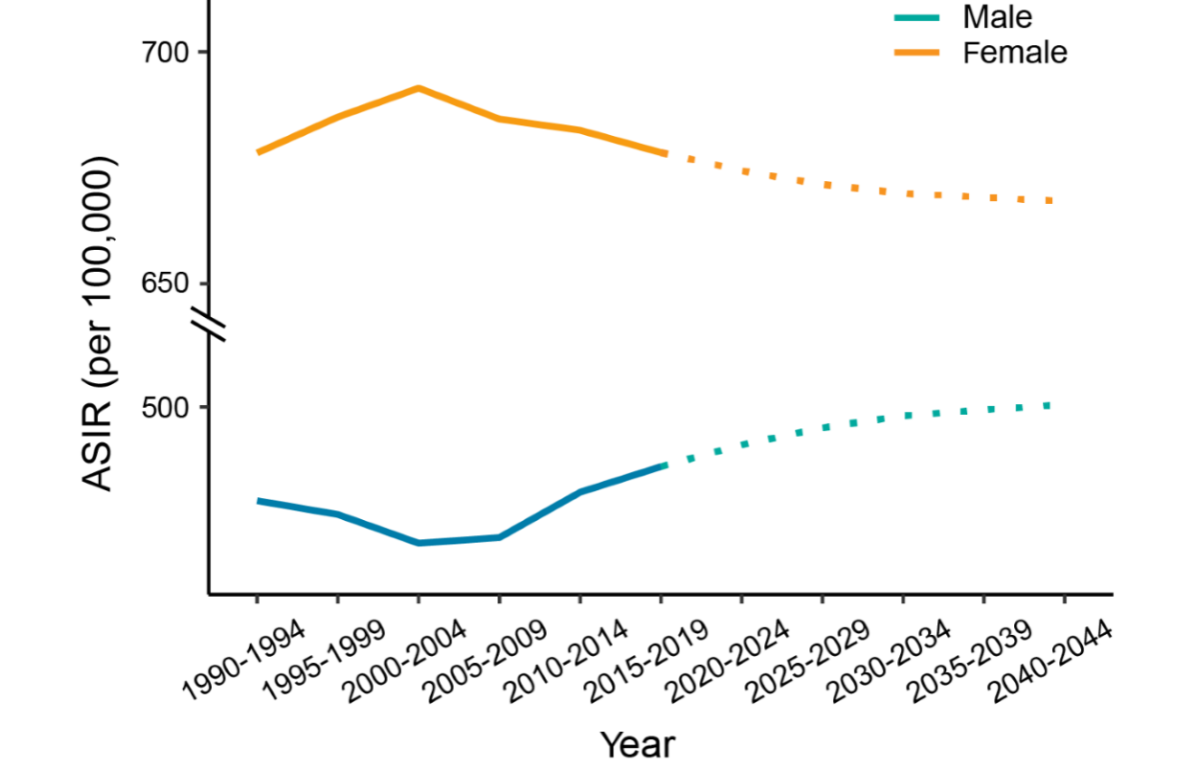
Predicted trends of anxiety disorders in 2020 to 2044: observed (solid lines) and predicted rates (dashed lines). ASIR: age-standardized incidence rate.

We predicted that the future ASIR of ADs would be relatively stable, but the trend would be the opposite for male individuals compared to female individuals. However, the incidence among male individuals will increase, and the incidence among female individuals will gradually decrease (Figure 4).

## Discussion

### Principal Findings

ADs are some of the most common mental disorders, and their prevalence and disease burden are affected by population growth trends, socioeconomic level, the natural environment, and other factors [5]. Our research analyzed the long-term trend in the incidence and DALY rate of ADs globally; evaluated the potential effects of age, period, and birth cohort; and predicted the prevalence trends in the next 25 years. This study provides guidance for the development of effective prevention and control policies.

In 1990, the CIR of ADs was 581.81 per 100,000, and it increased to 592.20 per 100,000 in 2019 (an increase of 1.79%). The DALY rate in 1990 was 348.81 per 100,000, and it increased to 370.61 per 100,000 in 2019 (an increase of 6.25%). This trend shows that Ads are still a global public health issue that cannot be ignored and that we need to strengthen the prevention and timely diagnosis and treatment of affected populations.

Our research showed that compared with the increase in the CIR and DALY rate, the increase in the ASIR of Ads was smaller, and the ASDR was slightly lower from 1990 to 2019. JPR model analysis showed that the trends of ASIR and ASDR were consistent in both sexes. Generally, the incidence of Ads and their DALYs should be very similar because DALYs are years of life lost plus years lived with disability, and years of life lost only accounts for a very small proportion of Ads in terms of DALYs because Ads are not a fatal disease. Therefore, it was not surprising that the ASDR would be very similar to the ASIR. Notably, the ASIR and ASDR decreased substantially between 2000 and 2005 for both sexes. This downward trend may be due to the rapid development of the global economy and culture, the great improvement of medical standards, and the declining birth rate [20].

In terms of incidence, the ASIR was higher among female individuals than among male individuals in 2019. This was consistent with the results of previous studies [11,26]. In 2015, a systematic review of prevalence studies showed that female individuals were twice as likely as male individuals to have ADs [4], which indicated that more female individuals are affected by ADs than male individuals worldwide. A strong body of evidence implicates sex hormone fluctuations in female individuals as the major biological factor driving sex differences in anxiety risk [11,26,27]. The ASDR among female individuals was also higher than that among male individuals, which is in line with a higher prevalence among female individuals than among male individuals. In addition, mental health knowledge has increased considerably, and stigma has decreased in countries that allow male individuals to talk about their feelings, seek help, and overcome the “rule”: male individuals drink and female individuals become depressed or anxious [28]. However, the ASIR of ADs was relatively stable (AAPCs 0.012%) and the ASDR decreased (AAPCs –0.015%) among female individuals, while both the ASIR (AAPCs 0.068%) and ASDR (AAPCs 0.035%) increased among male individuals. This may reveal a potentially increasing burden of ADs among male individuals.

Age-period-cohort model analysis showed that the upward trend in AD incidence was affected by age, period, and cohort. Age is an important demographic risk factor, and adolescents and middle-aged individuals are at high risk of ADs, similar to an M-shaped curve. The period effect showed that the overall incidence rate showed a downward trend, while the DALY rate showed an upward trend. The incidence of ADs increased, while the DALY rate decreased by year of birth.

Regarding the age effect, the RR of AD incidence was the highest in the age group of 10-14 years for both sexes, and the RR of the DALY rate was highest in the age group of 15-19 years among male individuals and in the age group of 20-24 years among female individuals. These findings were supported by a previous review [29]. Studies have shown that the high incidence of ADs among adolescents is closely related to genetic factors [30,31], higher vulnerability caused by hormonal processes, and adverse childhood experiences (serious illness, parental separation and emotional maltreatment, and physical and sexual abuse) [32-35]. Furthermore, the risk of ADs incidence among male individuals aged 40-44 years and female individuals aged 35-39 years was similar to that among adolescents, and then the risk decreased with age. The high risk of middle-aged people may stem from occupational pressure, family relationships, economic burdens, physical status, and other factors [36-38].

The period effect is usually caused by a series of complex historical events and environmental factors. We observed upward trends in the risk of developing ADs over time among male individuals after 2004. Notably, the incidence of ADs among male individuals increased rapidly during 2005-2010 and continued to increase until 2019 according to joinpoint analysis. This may be due to environmental changes and a more rapid pace of life [39,40]. During the study period, the world experienced a bird flu pandemic, rising oil prices, repeated terrorist attacks, and more severe natural disasters [41-44]. However, the period effect showed that the incidence of ADs among female individuals decreased over time after 1999, which was consistent with the reduction in incidence from 2000 to 2005 in the JPR analysis. The decline in incidence among female individuals may be due to higher levels of education, higher socioeconomic status, and a relative decline in fertility [38,45-47]. Compared with the incidence rate, the RR of the DALY rate increased over time in both sexes. This trend may be explained by the higher lifetime prevalence of ADs and the low and limited treatment rates in the early stage, which makes most patients with early-onset ADs develop a persistent course [48,49]. Long-term persistence or repeated attacks aggravate the degree of the disease and are prone to comorbidities and physical diseases, which increase the disability rate of these patients in the later stage [15]. Studies have found a lack of associations between sociodemographic characteristics and AD persistence, such as socioeconomic status and level of education, and inconclusive results for other socioeconomic factors, such as sex and age, although these characteristics were repeatedly found to be associated with the onset and prevalence of ADs [5].

The cohort effect on the incidence of ADs revealed continuous upward trends in later birth cohorts for both male and female individuals. The possible reason was that the later birth cohorts faced greater pressures, such as environmental degradation, dwindling resources, and economic depression. In contrast, the DALY rate of ADs decreased with birth year. The reason may be that the latest birth cohort was better educated, had better health care, and was more aware of health and disease prevention than the earliest birth cohort.

The predicted trend of AD incidence in our study over the next 25 years is consistent with the current trend, with a downward trend among female individuals and an upward trend among male individuals. This trend calls for us to pay more attention to the risk of ADs, especially among male individuals, and formulate effective prevention and treatment policies to reduce the incidence of ADs and thus reduce the disease burden.

In summary, between 1990 and 2019, the incidence of ADs among male and female individuals worldwide was dynamic. Overall, the global incidence rate among male individuals increased significantly while that among female individuals decreased significantly. According to age-period-cohort analysis, the incidence of the age effect showed an M-shaped curve. That is, adolescents and middle-aged people had the highest incidence. In addition, the incidence of ADs among male individuals will continue to increase soon. In this regard, we should pay more attention to the prevalence of ADs among male individuals, provide greater intervention and treatment for female individuals, and identify adolescent and middle-aged risk groups to support the timely initiation of intervention measures and effectively reduce the disease burden of ADs. At the same time, the COVID-19 pandemic has heightened anxiety, increasing the incidence of ADs and their disease burden [50]. Therefore, multiple interventions should be used aggressively to alleviate anxiety and promote mental health during the COVID-19 pandemic [51].

### Limitations

This study has limitations. First, GBD data are modeled at a higher setting and are derived from the analysis of limited raw data. Nevertheless, the results of this study rely heavily on the outcome data of the GBD study 2019. Second, the age-period-cohort model is the mathematical method used, and it does not fully reflect the reality of the situation. In addition, the GBD data do not include groups that are transgender. Therefore, the results should be interpreted with caution.

### Conclusions

In summary, ADs are becoming more common in both male and female individuals, and the disease burden in male individuals has increased slightly, indicating that early prevention and effective control cannot be ignored. The incidence of the age effect showed an M-shaped curve, and the incidence of ADs in male individuals will continue to increase soon. This suggests that we should take active intervention measures, focusing on high-risk groups, and develop effective management and control policies to reduce GBD.

## Appendix

Multimedia Appendix 1 Joinpoint regression analysis of the sex-specific age-standardized incidence and DALY rate for ADs globally from 1990 to 2019. (A) ASIR for male individuals; (B) ASIR for female individuals; (C) ASDR for male individuals; (D) ASDR for female individuals. AD: anxiety disorder; DALY: disability-adjusted life-year; APC: average percentage change; ASDR: age-standardized DALY rate; ASIR: age-standardized incidence rate.

## Abbreviations

AAPC: average annual percent change
AD: anxiety disorder
APC: annual percent change
ASDR: age-standardized disability-adjusted life-year rate
ASIR: age-standardized incidence rate
CIR: crude incidence
DALY: disability-adjusted life-years
DSM-5: Diagnostic and Statistical Manual of Mental Disorder, Fifth Edition
GBD: Global Burden of Disease
ICD-11: International Classification of Diseases, 11th Edition
JPR: joinpoint regression
RR: relative risk
UI: uncertainty interval

## Data Availability

The data sets supporting the conclusions of this article are available in the Global Burden of Disease Data Tool repository.
