# Supplementary figures and images for: Global Trends in the Incidence of Anxiety Disorders From 1990 to 2019: Joinpoint and Age-Period-Cohort Analysis Study
Source: JMIR Public Health Surveill. 2024 Jan 29;10:e49609. doi: 10.2196/49609 (PMC10862248; doi:10.2196/49609)

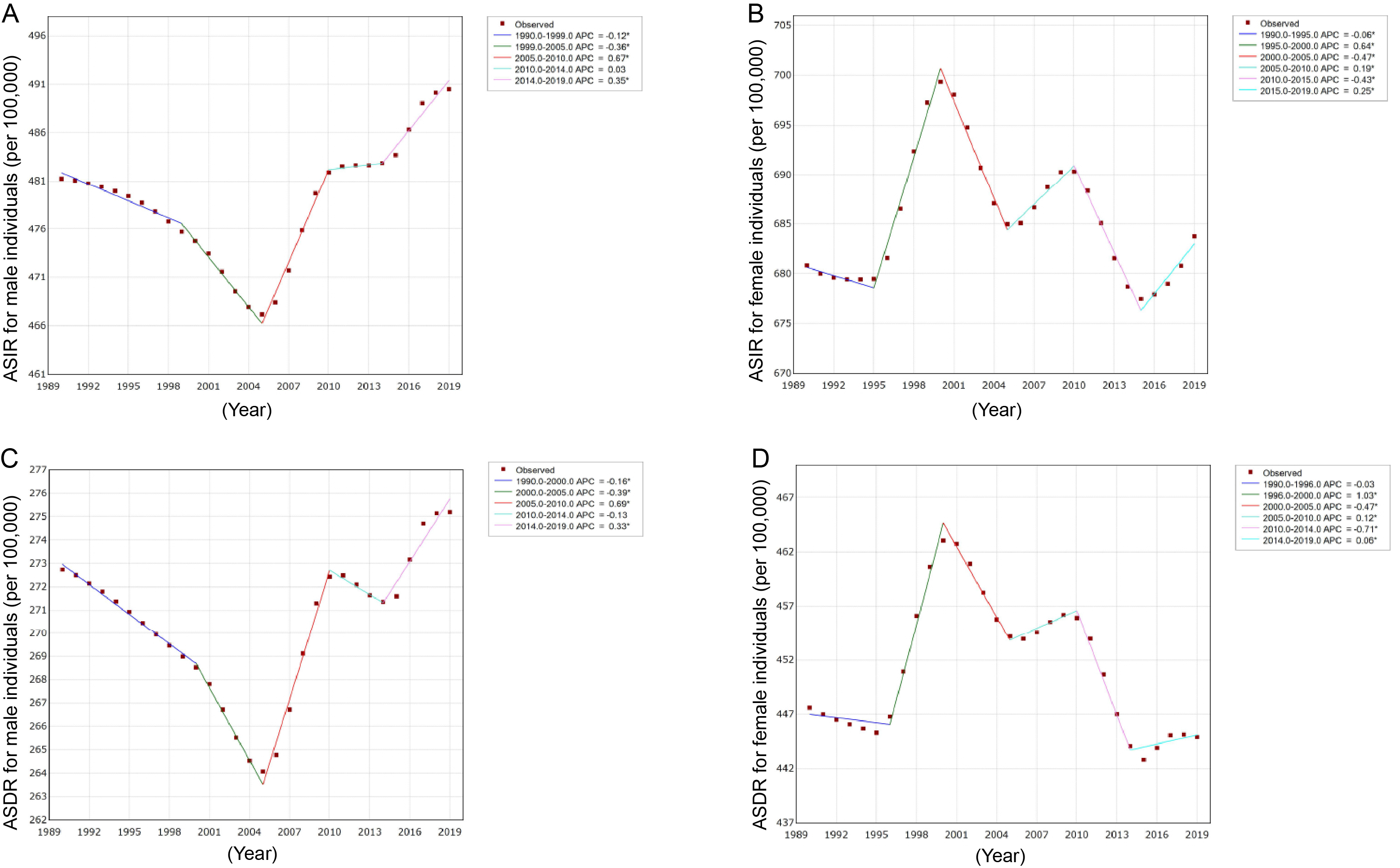

Supplement: Multimedia Appendix 1 [file publichealth_v10i1e49609_app1.png]
